# Immune Response in Crayfish Is Species-Specific and Exhibits Changes along Invasion Range of a Successful Invader

**DOI:** 10.3390/biology10111102

**Published:** 2021-10-26

**Authors:** Paula Dragičević, Dorotea Grbin, Ivana Maguire, Sofia Ana Blažević, Lucija Abramović, Anita Tarandek, Sandra Hudina

**Affiliations:** 1 Department of Biology, Faculty of Science, University of Zagreb, Rooseveltov Trg 6, 10000 Zagreb, Croatia; paula.dragicevic@biol.pmf.hr (P.D.); ivana.maguire@biol.pmf.hr (I.M.); sofia.ana.blazevic@biol.pmf.hr (S.A.B.); labramovic@stud.biol.pmf.hr (L.A.); atarandek@stud.biol.pmf.hr (A.T.); 2 Department of Biochemical Engineering, Faculty of Food Technology and Biotechnology, University of Zagreb, Pierottijeva Ulica 6, 10000 Zagreb, Croatia; dpolovic@pbf.hr

**Keywords:** immune response, crayfish abundance, water temperature, body condition, hepatosomatic index

## Abstract

**Simple Summary:**

A tiny fraction of all introduced species worldwide successfully establishes viable populations that rapidly spread and negatively impact native biodiversity and ecosystem functioning—i.e., become invasive. Even so, invasive species exert adverse environmental, economic, and human health impacts globally. Thus, identification of successful invaders is one of the immediate challenges in invasion biology. Recent studies highlight species’ immunity as an important component of invasion success since it enables invaders to adapt to the novel conditions as they expand their range. Here, we analyzed whether the immune response of the successful crayfish invader, the signal crayfish, changes as the invader encounters different environmental conditions and novel counterparts. We used several parameters, frequently applied to assess crayfish immunological status, and compared their cumulative pattern between sites along the signal crayfish invasion range in the Korana River, Croatia, as well as between the invasive signal crayfish and the native narrow-clawed crayfish. Immune response differed between native and invasive species and exhibited variations along the invasion range, which were mostly affected by water temperature and crayfish density. Our results indicate that changes in immunity may occur during range expansion, and imply that immunity could have a role in invasion success of invertebrate invaders.

**Abstract:**

Immunity is an important component of invasion success since it enables invaders’ adaptation to conditions of the novel environment as they expand their range. Immune response of invaders may vary along the invasion range due to encountered parasites/microbial communities, conditions of the local environment, and ecological processes that arise during the range expansion. Here, we analyzed changes in the immune response along the invasion range of one of the most successful aquatic invaders, the signal crayfish, in the recently invaded Korana River, Croatia. We used several standard immune parameters (encapsulation response, hemocyte count, phenoloxidaze activity, and total prophenoloxidaze) to: i) compare immune response of the signal crayfish along its invasion range, and between species (comparison with co-occurring native narrow-clawed crayfish), and ii) analyze effects of specific predictors (water temperature, crayfish abundance, and body condition) on crayfish immune response changes. Immune response displayed species-specificity, differed significantly along the signal crayfish invasion range, and was mostly affected by water temperature and population abundance. Specific immune parameters showed density-dependent variation corresponding to increased investment in them during range expansion. Obtained results offer baseline insights for elucidating the role of immunocompetence in the invasion success of an invertebrate freshwater invader.

## 1. Introduction

Invasive alien species (IAS) are characterized by fast dispersal, successful population establishment in a novel environment, and generation of negative effects on biodiversity, ecosystem structure, and functioning, as well as on human health and the economy [1,2,3,4]. Multiple studies suggest that the immune system of IAS is an important component of invasion success, since it is in a constant interaction with microbes/parasites and the environment during range expansion [5,6,7,8]. Therefore, variations in the immune response of invaders along the invasion range can be caused by both existing or novel parasites/microbial communities, local environmental conditions, and ecological processes during the range expansion [9,10,11,12]. For example, if during range expansion IAS encounter high pathogen and/or competitive pressure from congeners, or if environmental stress is high in the novel environment, IAS may redirect resources into the immune system instead of investing in processes that boost population growth and expansion [5,10,13,14].

Mounting the immune defense is energetically expensive, and thus IAS may use two possible strategies of immune response investment during range expansion [5]. The first one, a reduced investment in the immune response, assumes that the rapid dispersal of individuals at the invasion front will reduce the incidence of ‘enemies’ (e.g., parasites, pathogens, competitors or predators from their native range) within the population, especially if the first dispersers are healthier (i.e., less infected) and more fit [15]. Such a scenario would result in reduced pathogen presence at distribution edges, which is consistent with the enemy release hypothesis [16], and would allow for reduced energy investment in immunity and increased investment in life history traits promoting population growth and dispersal, i.e., faster individual growth and reproduction (evolution of increased competitive ability hypothesis (EICA)) [5,16,17,18]. In addition, the lower density of available hosts at the distribution edge (i.e., invasion front) may also potentially result in lower pathogen transmission rates and fewer immune challenges for hosts, which may further reduce the need for a strong immune response [7,15]. Conversely, the second strategy assumes an increased investment in the immune response during range expansion. At the invasion front, individuals are likely to come into contact with new unknown pathogens which may accumulate in the invader’s body (i.e., spillback hypothesis) [19], resulting in the need for increased investment in the immune response [5,20,21,22,23]. Furthermore, assuming that the dispersal of individuals is not random (i.e., spatial sorting theory) [24], and the less-infected individuals spread first, a better physiological status of such dispersers may indicate their higher immune potential [20,25]. Therefore, the stronger immune response at the invasion front could also appear as an indirect consequence of non-random dispersal of individuals in better condition [20]. Since dispersing individuals at the invasion front could benefit from both reduced and increased investment in the immune response, it is not immediately evident which of these opposing strategies will prevail during range expansion.

In order to investigate the changes in the immune response during range expansion, we selected one of the most successful IAS of freshwater invertebrates in Europe, the signal crayfish *Pacifastacus leniusculus* (Dana, 1852), which is listed among the Invasive Alien Species of Union concern (the Union list) according to the EU Regulation on invasive alien species No. 1143/2014. Invasive crayfish species have advantageous life history traits, such as fast growth, early maturation, high fecundity, and higher aggression in competitive interactions [26,27,28,29,30,31], which contribute to their rapid establishment and range expansion, and their negative impacts on freshwater ecosystems [32,33]. The population of signal crayfish selected for this research was first recorded in the Korana River, Croatia, in 2011 [34] and has been spreading successfully in both upstream and downstream directions ever since [35,36]. Differences between individuals along the invasion range have already been observed in this population by previous studies [37,38] and were similar to those described for other freshwater invaders (i.e., round goby) [39,40]: invasion fronts contained less aggressive individuals in better bodily and physiological condition, and females with better energetic status of hepatopancreas and gonads compared with the invasion core. Furthermore, the invasion range of the signal crayfish in the Korana River covers over 30 km of the watercourse [36], with segments potentially characterized by different environmental conditions: upstream (sparsely populated rural area) and downstream (industrial zone at outskirts of the Karlovac City). Considering the above-mentioned differences in fitness and their possible relation to immune potential [25], as well as potentially different environmental conditions along the Korana River, our goal was to examine whether differences in the immune response occur along the signal crayfish invasion range. 

Additionally, we compared the immune response of the invasive signal crayfish with the native narrow-clawed crayfish, *Pontastacus leptodactylus* (Eschscholtz, 1823). The narrow-clawed crayfish is a native species that is gradually increasing its range in the Korana and Mrežnica Rivers [41]. However, it has also been gradually outcompeted by the signal crayfish, and completely displaced from the signal crayfish invasion core [35,36]. The narrow-clawed crayfish co-occurs with the signal crayfish at the invasion fronts, where the signal crayfish populations are less abundant [35,36].

Like all invertebrates, crayfish lack adaptive immunity and rely upon the mechanisms of the innate immune system, such as melanin synthesis [42], coagulation system [43], and the production of antimicrobial peptides [44] as a response to parasite entry [45]. Melanin synthesized during crayfish immune response plays an important role in encapsulation of the microorganisms invading the hemocoel [42,46]. At the entry of an invading microorganism or foreign particle into the body, the prophenoloxidaze (proPO)-activating system is triggered. Hemocytes recognize the foreign particle (such as lipopolysaccharides, peptidoglucans, beta-1,3-glucans, i.e., parts of bacteria, fungi, etc.), which leads to the aggregation of other hemocytes and formation of a capsule surrounding the foreign particle (i.e., encapsulation) [42,47]. Simultaneously, proPO, the inactive precursor of phenoloxidaze (PO), is released from the hemocytes (granulocytes and semigranulocytes) into the hemolymph by exocytosis, where it is transformed into its active form (i.e., PO) by the serine protease [48,49]. The PO then catalyzes the synthesis of melanin, which is deposited in the capsule, resulting in capsule hardening, isolation of the foreign particle from the rest of the body, and infection localization [47,49,50,51]. In light of these processes, we measured the immune response in signal and narrow-clawed crayfish by using several standard immune parameters: strength of encapsulation response, total number of hemocytes in the hemolymph, PO activity, and total proPO. Activation of PO causes a drop in the level of total proPO [52], and indicates that there is a currently active, ongoing immune reaction in the individual. At the same time, the number of hemocytes drops because they are mobilized for the processes of encapsulation, coagulation, and/or degranulation in order to release more proPO into the hemolymph [53]. Consequently, the strength of encapsulation response (i.e., the level of melanization) measured at the site of infection should be proportional to the PO activity, and inversely proportional to the total proPO levels and number of hemocytes.

We aimed to (i) explore and compare the immune response of the native and invasive crayfish in their mixed populations by using the abovementioned immune parameters and (ii) to investigate in more detail the potential changes in the immune response of the expanding signal crayfish invader by comparing the immune response in individuals from populations of different age and crayfish abundance (invasion core and invasion front), as well as between individuals from upstream and downstream segments of the Korana River.

## 2. Materials and Methods

### 2.1. Study Area

The fieldwork was conducted in the Korana River, a 134 km-long karstic river located in continental Croatia, belonging to the Sava River basin. The study area covers approximately 33 km of the lower watercourse of this river, where the signal crayfish is spreading in both upstream (through the sparsely populated rural region) and downstream directions (through the industrial zone of the Karlovac City) [36]. The crayfish were sampled along the study area at sampling sites differing in crayfish community composition and crayfish abundance: (i) the invasion core sites which are characterized by longer-established, dense populations of the signal crayfish, and (ii) the invasion front sites which include recently established, less abundant signal crayfish populations, which co-occur with the native narrow-clawed crayfish [35,36]. Invasion front sites had 4 to 7 times lower relative total crayfish abundance (native narrow-clawed crayfish and invasive signal crayfish) in comparison with invasion core sites [36]. Invasion core sites contained no native crayfish, since they were outcompeted by the signal crayfish [35,36].

### 2.2. Sampling Procedure

The sampling was performed during the period of increased crayfish activity of both sexes of the signal and the narrow-clawed crayfish (i.e., before the mating period) [54], in the early autumn of 2020. The crayfish were captured at four sites along the lower reaches of the Korana River: upstream invasion front (UF), upstream invasion core (UC), downstream invasion core (DC), and downstream invasion front (DF; Table 1), previously identified by [36]. Only adult crayfish were caught using baited LiNi traps [55]—approximately 30 traps per site were left in the water overnight. Water temperature was measured at each site at the time of crayfish capture. Both signal crayfish individuals (captured at invasion cores and fronts, N = 138) and narrow-clawed crayfish individuals (captured only at invasion fronts, N = 13) of both sexes were used in the following analyses. Catch per unit effort (CPUE; i.e., equal to the number of crayfish captured per LiNi trap per trapping night) was calculated for each site based on the collected data. CPUE is a frequently used measure of relative crayfish abundance [56] and was used for population abundance comparisons among sites.

### 2.3. Immune Response Analyses

Immune response analyses included several standard immune parameters which are frequently analyzed in crustaceans: (i) the strength of the encapsulation response, i.e., the amount of synthesized melanin [57,58], (ii) total hemocyte count (THC) [59,60,61,62,63], and (iii) enzyme activity of phenoloxidaze (PO) and total prophenoloxidaze (proPO) [42,61,64,65]. These four parameters were analyzed together as the immune response of crayfish.

#### 2.3.1. The Encapsulation Response Analyses

The experimental immune challenge was conducted on a total of 126 captured signal crayfish individuals (approximately 30 at each sampling site: UF, UC, DC, and DF; Supplementary Table S1), and a total of 13 captured narrow-clawed crayfish individuals (captured at both invasion fronts, i.e., UF and DF; Supplementary Table S2A). A sterile nylon monofilament implant method was used immediately upon capture in the field to induce the encapsulation response and to obtain a standardized measure of the encapsulation response strength, which is strongly related to the defense against parasites [66,67,68,69]. A nylon monofilament (i.e., fishing line, Jaxon Satori, Japan; from here on referred to as implant) was roughened with sandpaper, tied into a knot, and cut to the desired length under the knot. Prior to insertion, the implants (4 mm long, 0.22 mm in diameter) were stored in 90% ethanol to ensure sterility. Implants, representing novel and standardized pathogens, were inserted through a small puncture in the first joints of each of the fifth pair of walking legs using forceps [57,58]. Each individual was then placed in a perforated plastic container (18 × 18 × 9 cm; with numerous perforations approximately 0.7 cm in diameter) that allowed water circulation. Containers with crayfish were then submerged in the river at the exact site where crayfish were caught and left for 48 h. After the 48 h period, the crayfish in containers were put on ice and taken to the laboratory for implant extraction, measurement, and hemolymph sample collection.

In the laboratory, the implants were retrieved from individuals’ walking legs using forceps and stored at −20 °C. In further analyses, the two implants from walking legs of each crayfish individual were placed on a white background along with a sterile implant and photographed from two different sides using a digital camera connected to a light microscope (Stemi 305, Zeiss, Germany). In order to quantify the strength of the encapsulation response (i.e., the degree of melanization), the image-processing program (ImageJ, ver. 1.53f, https://imagej.nih.gov/ij/index.html, accessed on 3 Nov 2020) was used to determine the gray values of reflecting light of the melanized implants [57,58]. Encapsulation response strength was determined by subtracting the mean of the two gray values of a melanized implant from the gray value of a sterile (clear) implant [66]. Finally, the encapsulation response strength per individual was determined by calculating the mean gray value of both inserted implants.

#### 2.3.2. Hemolymph Sampling Procedure

Following implant removal, the individuals were measured (total length (TL), length of the postorbital part of the carapace (POCL)) was weighed, and their hemolymph was sampled. Using a sterile needle, minimally 500 µL of hemolymph was collected from the base of the individual’s walking leg, of which: (i) 100 µL was diluted in 400 µL of 1% formalin for total hemocyte count (THC), and (ii) 400 µL was diluted in 800 μL of crayfish saline solution (CFS: 0.2 M NaCl, 5.4 mM KCl, 10 mM CaCl_2_, 2.6 mM MgCl_2_, 2 mM NaHCO_3_, pH 6.8) [48] for the analyses of PO activity and total proPO. The hemolymph samples collected for PO and proPO analyses were immediately centrifuged at 10,000× *g* for 10 min at 4 °C to prevent coagulation. Next, they were put on ice and sonicated for 10 s with gradually increasing power to 50% (Sonoplus HD 2070, Bandelin, Germany) in order to completely lyse the hemocytes and release the stored proPO into the diluted plasma. Finally, the samples were centrifuged again at 15,000× *g* for 25 min at 4 °C to separate cell debris, and the supernatant was stored at −80 °C. After hemolymph sampling, each individual was killed according to available guidelines for humane killing of crayfish (rapid cut of nerve cord from thorax to the end of abdomen; however, no institutional or national ethical guidelines exist for crayfish) [70]. Each animal was then dissected, and their hepatopancreases were carefully removed and weighed for the analyses of body condition parameters (described below).

#### 2.3.3. Total Hemocyte Count

The hemolymph samples collected for THC were stored at 4 °C for hemocyte fixation until further analyses. The hemocytes were counted by using the Bürker-Türk counting chamber and Zeiss Standard RA Light Microscope [71,72]. The number of hemocytes per milliliter was calculated after taking into account the dilution of the hemolymph during sampling [72,73].

#### 2.3.4. PO Activity and Total proPO

From each individual, hemolymph concentrations of active PO and total proenzyme proPO were measured spectrophotometrically in supernatant samples (containing both active PO and inactive proPO released from the hemocytes) prepared in the previous step, following a modified version of the method by [74]. Briefly, to measure the PO activity, 50 µL of the L-3,4-dihydroxyphenylalanine substrate (L-DOPA; 3 mg/mL, dissolved in Milli-Q water) were mixed with 50 µL of each sample in a microplate (in triplicates), and the absorbance was measured at 490 nm for 25 min. In order to quantify the proenzyme proPO, all the available proPO in the samples first had to be converted into their active form (enzyme PO). Therefore, in another spectrophotometrical assay, 50 µL of each sample were preincubated with 50 µL of trypsin (acting as an elicitor; 1 mg/mL, dissolved in Milli-Q water) in a microplate for 3 min at room temperature. Afterwards, 50 µL of L-DOPA were added to the reaction mix and the absorbance was measured at 490 nm for 25 min again. The amount of total proPO in samples was calculated as total proPO measured in the trypsin treatment minus the PO activity measured before trypsin treatment [52,74,75]. Finally, in order to standardize the enzyme activity per mg of protein [46], total protein content was measured using the method by [76], as in [77]. Enzyme activity was expressed as the change in absorbance at 490 nm per min and mg of protein (ΔA_490_/min/mg protein).

### 2.4. Body Condition Parameters

Since immune response is considered an important fitness component [78] and dependent upon animal (physiological) condition, we measured several condition parameters in addition to immune parameters: (i) Fulton’s condition factor (FCF = W/TL^3^ × 100; where W = weight (g), and TL = total body length (mm) of the individual), which is used as a proxy for individual’s body condition [79], and (ii) hepatosomatic index (HSI = HW/BW; where HW = hepatopancreas weight (g), and BW = body weight (g)), which is indicative of an individual’s energy status [80]. These indices are frequently used to determine health and fitness of crayfish individuals [37,81,82,83,84] and are also used as proxy measures of fitness in aquatic animals [85].

### 2.5. Statistical Analyses

#### 2.5.1. Comparisons of Changes of the Signal Crayfish Immune Response along Invasion Range and Their Potential Drivers

To explore the effects of specific predictors on the changes in the signal crayfish immune response, the Partial Least Squares Regression approach (PLS-R) was used. In the present study, the explanatory variables (predictors, X) were water temperature, relative crayfish abundance (i.e., CPUE), and crayfish condition indices (FCF, HSI), while the response variables (Y) were measured immune parameters (encapsulation response, THC, PO activity, and total proPO). The PLS scores associated with the first two PLS components, generated in the model, are new variables summarizing the X variables. Scores contain the information about the objects and their similarity [86] and were therefore used for the interpretation of the PLS-R model. We reported model quality indices Q^2^(cum), R^2^Y(cum), and R^2^X(cum) parameters and calculated standardized coefficient to examine how changes in predictors (water temperature, CPUE, FCF, HSI) affect response variables (immune response: encapsulation response strength, THC, PO activity, total proPO) and which predictors have a greater effect on the response variables. Additionally, in order to examine which of the predictors have the highest explanatory power for the construction of the immune response, we performed a variable importance for the projection (VIP) procedure. Parameters with a VIP value > 1 were considered relevant for explaining the response variables (Y) and contributed significantly to the model, while parameters with a VIP value < 0.8 contributed little [87,88,89]. Furthermore, we performed generalized linear model (GLM) analysis fitted with aov function on PLS scores to test for the significance in the relationship between response variables and predictors towards sites along the invasion range (DF, DC, UF, UC), upstream (UF, UC) and downstream (DF, DC) river segments, invasion core (UC, DC) and invasion front (UF, DF) sites, and sex. Analyses were performed using statistical software R v. 3.6.2 [90]. Exceptionally, the PLS-R analysis was partly performed using the “plsdepot” package according to [91] in statistical software R, and partly using the XLSTAT version 2018.3 software for data analysis and visualization of radar of correlation provided by Microsoft Excel by Addinsoft. The “ggbiplot” package [92] in R was used for visualization of the PLS-R score plots and principal component analysis (PCA) biplot, while basic R “stats” package was used to perform GLM on PLS scores. In all analyses, the significance threshold was set at *p* < 0.05.

#### 2.5.2. Comparisons of Immune Response between the Invasive Signal Crayfish and the Native Narrow-Clawed Crayfish

PCA was used for comparison of immune response between the two species (invasive signal crayfish and native narrow-clawed crayfish) from their mixed populations at invasion fronts in order to illustrate the importance of immune variables (i.e., encapsulation response strength, THC, PO activity, and total proPO) for the separation of the species. For this analysis, signal crayfish individuals were selected from the pool of all collected individuals (Supplementary Table S2B) so that the sex ratio and body size were kept similar between the species, and were compared to the collected narrow-clawed crayfish individuals (Supplementary Table S2). To test for the significance of the influence of the immune variables in species separation, a GLM fitted with aov function was performed on PCA scores (using basic R “stats” package). A threshold of *p* < 0.05 was considered significant.

## 3. Results

### 3.1. Comparisons of Changes of the Signal Crayfish Immune Response along Its Invasion Range and Their Potential Drivers

**Figure 1:**
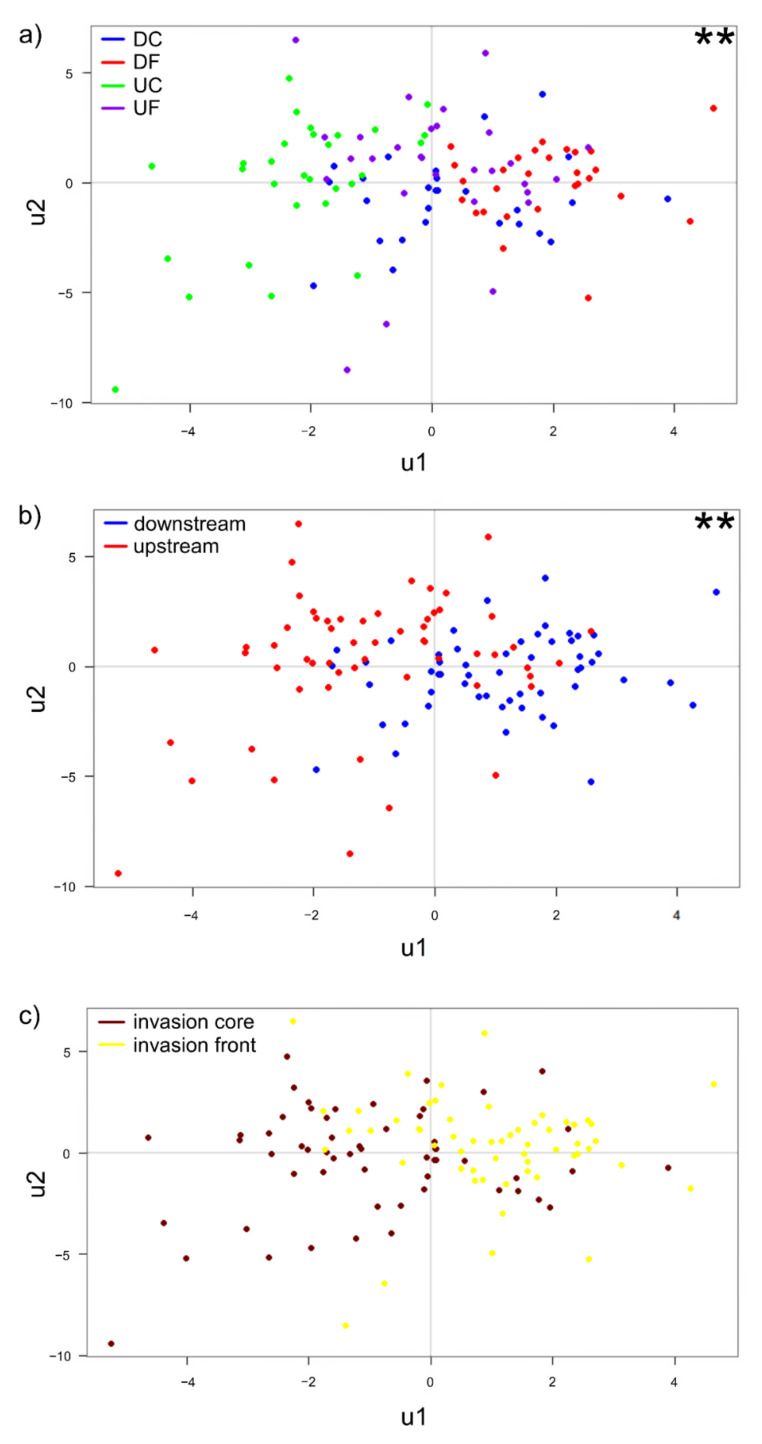
Partial least squares regression (PLS-R) score plots of signal crayfish immune parameters, based on y components (u1 and u2). Plots represent the relationship between response variables (immune parameters) and predictors (water temperature, relative crayfish abundance (CPUE), Fulton’s condition factor (FCF), and hepatosomatic index (HSI)) according to sites along invasion range (**a**), upstream and downstream river segments (**b**), and invasion core and front sites (**c**). Significant effect (*p* < 0.01) in ‘response-predictor’ relation (performed by using the generalized linear model on PLS-R scores) is indicated with **. DC = downstream invasion core, DF = downstream invasion front, UC = upstream invasion core, UF = upstream invasion front.

Using the PLS-R multivariate technique, the relationship between the immune parameters and specific predictors, i.e., water temperature, relative crayfish abundance (CPUE), Fulton’s condition factor (FCF) and hepatosomatic index (HSI), was determined. The results of the GLM (Table 2) showed that, based on specified predictors, immune parameters significantly differed (*p* < 0.05) between sites along the invasion range and between upstream and downstream river segments (Figure 1a,b, Table 2). However, the immune parameters did not exhibit significant separation between invasion core and front sites (Figure 1c, Table 2). No clustering was observed for sexes, showing no difference in immune response between males and females among or within all inspected groups (Table 2).

In the PLS-R model, the first component was calculated with the Q^2^(cum), R^2^Y(cum), and R^2^X(cum) parameters of 0.12, 0.13, and 0.42, respectively and the second component was calculated with the Q^2^(cum), R^2^Y(cum), and R^2^X(cum) parameters of 0.14, 0.17, and 0.54, respectively (Supplementary Figure S1). 

**Figure 2:**
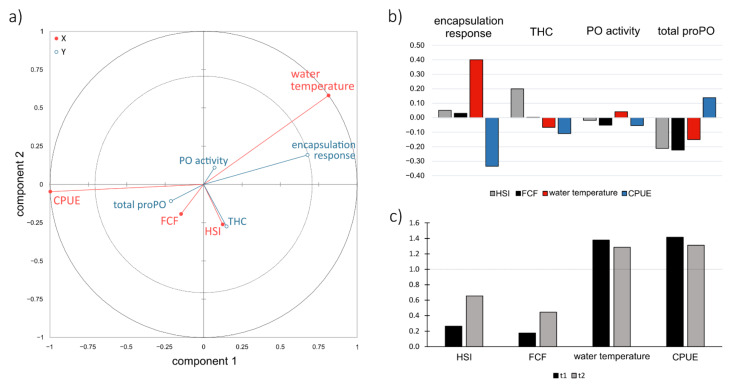
(**a**) Radar of correlation, illustrating the relationship between response variables (signal crayfish immune parameters, represented with blue lines) and predictors (represented with red lines). (**b**) Standardized coefficients of signal crayfish immune response. The closer to the absolute value of 1 the coefficient is, the stronger the effect of that predictor on the response variable (controlling for other variables in the equation). (**c**) Variable importance for the projection (VIP) for explanatory variables of first two components (t1 and t2). VIP > 1 indicate the explanatory variables that contribute the most to the PLS model. CPUE = catch per unit effort (relative crayfish abundance), FCF = Fulton’s condition factor, HSI = hepatosomatic index, PO = phenoloxidaze, proPO = prophenoloxidaze, THC = total hemocyte count.

The relationship between blocks of predictor and response variables is visually presented in the form of a radar of correlation (Figure 2a), where positively correlated variables are presented close to each other and for negative correlation, variables are located far from one another. PLS-R multivariate analysis showed that encapsulation response strength had the strongest correlation with temperature (*r* = 0.66) and relative crayfish abundance (CPUE; *r* = −0.68), while other immune parameters exhibited measurable to moderate correlations in predictor-response relation (Figure 2a; Supplementary Table S3). Phenoloxidaze (PO) activity exhibited a similar pattern of correlation as encapsulation response strength (positive correlation with water temperature and negative correlation with relative crayfish abundance: *r* = 0.12 and *r* = −0.07, respectively), while total proPO exhibited an inverse correlation pattern (water temperature: *r* = −0.24; CPUE: *r* = 0.22; Figure 2a). Standardized coefficients (Figure 2b) also showed that both water temperature and CPUE had a stronger effect on immune response than crayfish condition in the case of encapsulation response strength and PO activity, while total hemocyte count (THC) and total proPO were more influenced by the crayfish condition: HSI in the case of THC (*r* = 0.16) and both HSI and FCF in the case of total proPO (*r* = −0.21, *r* = −0.1, respectively). Additionally, water temperature and CPUE generally exhibited the highest explanatory power for the construction of the immune response (Figure 2c).

### 3.2. Comparisons of Immune Response between the Invasive Signal Crayfish and the Native Narrow-Clawed Crayfish

**Figure 3:**
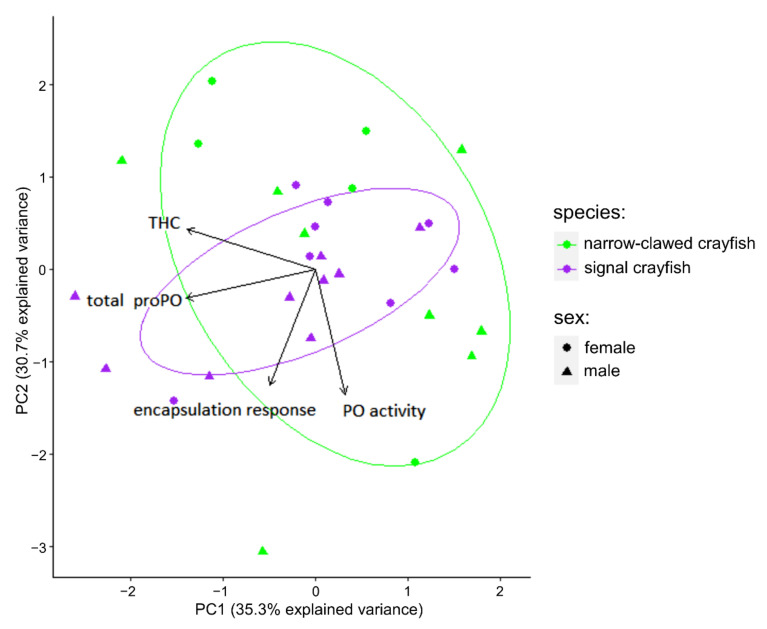
Principal component analysis (PCA) biplot on immune parameters of the signal crayfish and narrow-clawed crayfish from their mixed populations. Variables are indicated by black arrows, where their length represents the influence of a specific variable in shaping a model. 95% confidence ellipses are estimated around clusters. PO = phenoloxidaze, proPO = prophenoloxidaze, THC = total hemocyte count.

PCA of immune parameters between the two crayfish species revealed that the first two principal components explain 66% of the total variance, PC1 = 35.3% and PC2 = 30.7% (Figure 3). The biplot (Figure 3) shows the relationship between immune parameters. If the angle between the two variable vectors is zero, then it shows both variables are collinear. Here, results demonstrated that encapsulation response correlated the most with PO activity, while THC correlated with total proPO. The results of the generalized linear model (GLM; Table 3) showed significant separation (*p* = 0.006) between the two crayfish species according to immune response variables. No clustering was observed for sexes, showing no difference in immune response between males and females within each species. Further, PC loadings (Supplementary Table S4) on the first two PC’s showed that all analyzed variables contributed in a very similar proportion to species separation (graphically presented by biplot obtained on the first two principal components, black arrows on Figure 3).

## 4. Discussion

Crayfish immune response is a result of complex interactions of multiple intrinsic (e.g., body condition, parasite load, diseases) and extrinsic (e.g., environmental conditions, population density, predation risk) factors [93,94,95]. In addition to these factors, the invasion process may also affect the immune response of both the crayfish invader and the native crayfish species due to potential trade-offs between immunity and the host’s reproductive fitness [7,96,97] and potential spatial sorting of individuals with certain life-history traits during non-random dispersal (i.e., [98]). Here, we analyzed the differences in the immune response of the invasive signal crayfish along its invasion range. Additionally, we examined whether the immune response in crayfish is predominantly determined by intrinsic (body condition: hepatosomatic index (HSI), Fulton’s condition factor (FCF)) or extrinsic (water temperature, relative crayfish abundance (CPUE)) factors. Finally, the immune response of the invasive signal crayfish was compared to that of the native narrow-clawed crayfish, a species negatively affected by the signal crayfish range expansion. As the immune system of IAS may affect their invasion success [21], our results contribute to better understanding of the factors determining the immune response during invasive species’ range expansion.

### 4.1. Comparisons of Changes of the Signal Crayfish Immune Response along Its Invasion Range and Their Potential Drivers

Clear differences observed between all four signal crayfish populations (UF, UC, DC, DF) indicate that, based on the given predictors in PLS-R analysis, the immune response changes significantly along species’ invasion range in the Korana River. Significant differences in the immune response were also established between upstream and downstream populations, but not between the invasion core and invasion front populations, suggesting that variation in the immune response resulting from range expansion may be outweighed by the effects of the local (abiotic) environmental factors, which may present a more prominent driver of changes in the immune response of crayfish. Our results are congruent with other studies [99,100] which examined the immune and glucocorticoid responses of invasive cane toads and found no differences in individuals from invasion core and invasion front populations, while multiple other studies report that immune response may show variation between the invasive species populations of different age and relative abundance along the invasion range [12,101,102,103].

The observed significant differences in immune response between the upstream and downstream signal crayfish populations might be due to differences in microhabitat characteristics. The approximately 33 km-long invasion range of the signal crayfish in the Korana River consists of microhabitats which differ in temperature, surrounding vegetation, water depth, sediment type, and anthropogenic pressure, with the upstream part of the Korana River flowing through the sparsely populated rural region, and the downstream part of the invasion range located in the industrial zone of the Karlovac City [104]. Additionally, it has been previously reported that different environmental parameters (e.g., temperature, pollution, oxygen levels, pH, and salinity) may affect crayfish immune system/health status [93,105,106,107,108,109,110]. In the case of the Korana River, similar water quality status in terms of general physical and chemical conditions and specific pollutants has been previously recorded across all seven water body monitoring sites monitored according to Water Framework Directive [111], which also cover the whole signal crayfish invasion range within the river (Water Body Register 2016–2021; Croatian Waters). However, we recorded differences between upstream and downstream river segments in water temperature, which was 5.6 °C higher at downstream compared with upstream river segments. Temperature has been shown to exert variable and opposing effects on crustacean immune response in different studies [61,105,106,108,112,113] and has been suggested to have a prominent role in arthropod immune response along with population density [114,115,116,117,118,119,120,121,122]. This was corroborated by our study, where out of the predictor variables, water temperature and the relative crayfish abundance (CPUE) had much higher explanatory power of immune response in comparison with body condition parameters, and were considered the most relevant to the immune response construction.

### 4.2. Relationships between the Predictors and Immune Parameters

Stronger encapsulation response, i.e., higher level of melanization, is a result of an enhanced PO activity [49]. Since PO is released from the semigranulocytes and granulocytes by degranulation, this process leads to a drop in the number of hemocytes [53]. However, after the initial reaction to infection in the form of dramatic hemocyte depletion, the hematopoietic tissue is stimulated to rapidly synthesize and release new hemocytes [123,124]. Here, we found that total hemocyte count (THC) was the only immune response parameter which showed a negative correlation with both water temperature and relative crayfish abundance, suggesting that the exposure to environmental stress may lower the number of hemocytes [53,125,126], since hemocytes represent the first line of defense in the crayfish immune system and participate in the processes of immediate immune reactions (i.e., clotting, melanization, phagocytosis, encapsulation) and act as suppliers of antimicrobial peptides, lectins, proteinase inhibitors, and opsonins [51].

The encapsulation response strength and phenoloxidaze (PO) activity were positively correlated with water temperature, indicating that a higher level of melanization occurs as a stress response to higher environmental temperatures. Another study [118] reports similar findings in exploring the effects of temperature and population density on the immune response of the arthropod velvetbean caterpillar, suggesting that temperature is the main environmental factor affecting the host immune defense. Total protein content in the hemolymph (including PO) is dependent upon multiple factors, such as species, molting, reproduction, nutritional state, infection, stress response, salinity, season, light period length, temperature, and the level of dissolved oxygen [127,128]. The latter is directly related to water temperature (i.e., level of dissolved oxygen decreases with increasing water temperature). Therefore, changes in water temperature, which are manifested through changes in available oxygen, can directly or indirectly affect PO-specific activity (i.e., measured immune parameters). Furthermore, similarly to our results, [121] reported a temperature-dependent increase in capsule melanization (i.e., encapsulation response). However, mounting a strong encapsulation response may also negatively affect the crayfish due to self-reactivity costs of a strong immune defense [57,129] since the process of melanin synthesis during the encapsulation response also involves the release of cytotoxic byproducts, such as quinones and phenols, which damage the tissue [130].

Both encapsulation response strength and PO activity were negatively correlated with the relative crayfish abundance, which suggests that the encapsulation response strength (and PO activity) is higher in individuals from populations of low relative crayfish abundance (i.e., invasion front). Obtained results are further corroborated by the pattern of correlation observed for total prophenoloxidaze (proPO), which showed an inverse relationship compared with the above parameters (negative correlation with water temperature, and positive correlation with the relative crayfish abundance). This is consistent with our expectations that the decrease (activation) of proPO is followed by the increase in PO activity, and increase in the level of melanization (i.e., encapsulation response strength). Thus, while an overall immune response seemed not to be affected by the dispersal process (i.e., showed no significant differences between invasion core and invasion front populations), specific immune parameters showed density-dependent variation corresponding to increased investment in them during range expansion. Such conflicting effects have already been previously reported [100], and are not surprising since: i) density influences both intraspecific competition and pathogen transmission rates, and ii) the animal immune system is multifaceted and complex, and its different components may exhibit different patterns of change [101].

Despite an overall low explanatory power, both THC and total proPO were affected by crayfish condition (FCF: total proPO and HSI: total proPO and THC). However, the relationship between crayfish condition parameters and immune parameters is still largely unexplored. The hepatopancreas is a central organ of crustacean immunity and metabolism, as well as the main energy storage that supports key functions such as reproduction and growth [131,132], and organosomatic indices are considered as viable proxy measures of fitness [85,133]. Immunity is also an important fitness component, since it enables the organism to fight its pathogens, survive, and reproduce (i.e., [134]). Thus, the relationship between energy status of hepatopancreas (measured by organosomatic indices) and additional fitness determinants (such as, for example, reproductive success) and immune parameters should be further examined. However, this is a complex task since quantification of different fitness components/determinants needs to take into account the effects of the season, year cycle characteristics related to molting and mating, presence of an acute or chronic infection, etc. [135,136]. Finally, in order to elucidate the role of immunocompetence in invasion success, further studies involving examination of additional parameters related to both crayfish status (i.e., fecundity, individual health status), as well as environmental conditions (i.e., detailed ecological and chemical status), should be conducted. Additionally, future studies should also be performed at multiple locations (i.e., rivers, lakes) with recorded signal crayfish presence and over a longer time period.

### 4.3. Comparisons of Immune Response between the Invasive Signal Crayfish and the Native Narrow-Clawed Crayfish

Significant differences in the immune response were observed between the invasive and the native crayfish species in the Korana River, even though they inhabit the same local environment (i.e., co-occur at the same sites with same water temperature and relative crayfish abundance). This clearly shows the species-specific differences related to the measured immune parameters. Previous studies have shown that signal crayfish, the original host of the pathogen *Aphanomyces astaci* Schikora, 1906, has adapted to its presence in the body and is able to carry the latent infection by keeping the immune defense at a constantly high level [137,138]. The resistant invasive signal crayfish had continuously elevated levels of proPO expression, which could not be additionally increased by immunostimulants like in a susceptible native crayfish species [138], such as the narrow-clawed crayfish. The prevalence of crayfish plague in the signal crayfish population in the Korana River was very low (6% of individuals, distributed equally along the invasion range) [139], while qPCR quantification of *A. astaci* performed in parallel with this study identified very low agent levels (A0–A3, with the majority of samples from individuals of both species classified as A0; Bielen et al., in preparation). Therefore, neither native nor invasive crayfish populations show signs of recent crayfish plague outbreaks, which could potentially have a high impact on their immune response. Even though all immune parameters contributed similarly to the separation between species in PCA analysis, differences in proPO expression may represent the main driver of the observed differences in the immune response between the two species in this study, since they consequently affect all immune parameters measured here (as elaborated in the Introduction). However, immune response to *A. astaci* infection is much more complex than the activation of the proPO cascade, which makes only a small portion of crayfish humoral response [140]. Therefore, further studies are required to elucidate the mechanisms and energetic costs of mounting an immune response in invasive and native species differing in the proPO expression. Comparisons of energetic costs of immune response between invasive and native crayfish in pathogen-free and infected populations are required in order to clarify this question.

## 5. Conclusions

In conclusion, we observed differences in the immune response along the invasion range, with the environmental and population characteristics (water temperature and population density) being the more prominent drivers of changes in the immune response compared with the invasion process, whose impact on the immune system was not evident in this study (i.e., no significant differences in immune response were observed between invasion core and invasion front populations). While the overall immune response seemed not to be affected by the dispersal process, specific immune parameters showed density-dependent variation corresponding to an increased investment in them during range expansion. Furthermore, since the relationships between immune parameters were not as distinct as expected, further research is required to clarify the cause-and-effect relationship with animal condition and/or environmental factors (such as the season and year cycle characteristics related to molting and mating, temperature, presence of an acute or chronic infection, etc.). Finally, we confirmed that the immune response is species-specific, exhibiting significant differences between the co-occurring native narrow-clawed crayfish and invasive alien signal crayfish. The obtained results represent the first step in investigating the role of immunocompetence in the invasion success of an invertebrate freshwater invader, required for elucidating the costs of immunity and its links to individuals’ reproductive success and overall fitness.

## Figures and Tables

**Table 1:** Geographic coordinates of sampling sites along the invasion range of the signal crayfish in the Korana River in 2020.

Location	X (WGS84)	Y (WGS84)
upstream front (UF)	45.320915	15.518373
upstream core (UC)	45.371918	15.521505
downstream core (DC)	45.411808	15.609231
downstream front (DF)	45.451355	15.567030

**Table 2:** Generalized linear model (GLM) fitted with aov on PLS-R scores of immune parameters of signal crayfish. Significant differences (p < 0.01) are indicated with **. Df = degrees of freedom, Sum Sq = sum of squares, Mean Sq = mean squares.

MODEL	Df	Sum Sq	Mean Sq	F Value	*p* Value
**sites along invasion range**					
**sites**	3	625	208.24	4.37	0.006 **
**sex**	1	12	12.32	0.26	0.61
**sites:sex**	3	133	44.22	0.93	0.43
**residuals**	106	5052	47.66		
**downstream-upstream**					
**downstream-upstream**	1	410	409.9	8.35	0.004 **
**sex**	1	12	12.2	0.25	0.62
**downstream-upstream:sex**	1	0	0	0	0.99
**residuals**	110	5399	49.1		
**core-front**					
**core-front**	1	112	111.5	2.2	0.14
**sex**	1	2	2.38	0.05	0.82
**core-front:sex**	1	149	148.95	2.95	0.08
**residuals**	110	5559	50.5		

**Table 3:** GLM fitted with aov on PCA scores of immune parameters of two crayfish species. Significant differences (p < 0.01) are indicated with **. Df = degrees of freedom, Sum Sq = sum of squares, Mean Sq = mean squares.

MODEL	Df	Sum Sq	Mean Sq	F Value	*p* Value
**species**	1	23.6	23.59	9.91	0.006 **
**sex**	1	13.35	13.35	5.61	0.25
**species:sex**	1	0.1	0.1	0.04	0.83
**residuals**	26	61.91	2.38		

## Data Availability

The data that support the findings of this study are available from the corresponding author upon request.

## Supplementary Materials

The following are available online at https://www.mdpi.com/article/10.3390/biology10111102/s1, Figure S1: Model quality by number of components, Table S1: Number of signal crayfish per site included in the analyses of changes in the immune response along the species’ invasion range and their potential drivers, Table S2: Number of narrow-clawed (A) and signal crayfish (B) per front site included in the comparative analyses of immune response between the invasive and native species, Table S3: Correlation matrix of the analyzed variables, Table S4: Principal component loadings (PC1 and PC2) on immune parameters of the invasive signal crayfish and the native narrow-clawed crayfish.
