# Supplementary material for: Immune Response in Crayfish Is Species-Specific and Exhibits Changes along Invasion Range of a Successful Invader
Source: Biology (Basel). 2021 Oct 26;10(11):1102. doi: 10.3390/biology10111102 (PMC8615248; doi:10.3390/biology10111102)
Supplement: Supplementary file 1 [file biology-10-01102-s001.zip › biology-1414747-supplementary.pdf]

Table S1. Number of signal crayfish per site included in the analyses of changes in the immune response along the species' invasion range and their potential drivers.

signal crayfish (all individuals)					
Location	Total individuals	Males	Females	Average postorbital carapace length (mm)	Average body weight (g)
upstream front	30	15	15	46.59 (±5.69)	55.13 (±19.36)
upstream core	32	18	14	46.91 (±6.13)	46.74 (±19.41)
downstream core	32	11	21	46.33 (±5.67)	41.78 (±13.84)
downstream front	32	9	23	42.11 (±6.02)	32.8 (±15.27)

Table S2. Number of narrow-clawed (A) and signal crayfish (B) per front site included in the comparative analyses of immune response between the invasive and native species.

A narrow-clawed crayfish					
Location	Total individuals	Males	Females	Average postorbital carapace length (mm)	Average body weight (g)
upstream front	3	0	3	41.84 (±4.46)	30.94 (±10.59)
downstream front	10	8	2	45.73 (±5.33)	37.31 (±12.66)
B signal crayfish (individuals chosen for comparative analyses with narrow-clawed crayfish)					
Location	Total individuals	Males	Females	Average postorbital carapace length (mm)	Average body weight (g)
upstream front	6	0	6	40.80 (±3.58)	36.69 (±5.89)
downstream front	12	7	5	40.98 (±5.02)	30.69 (±12.67)

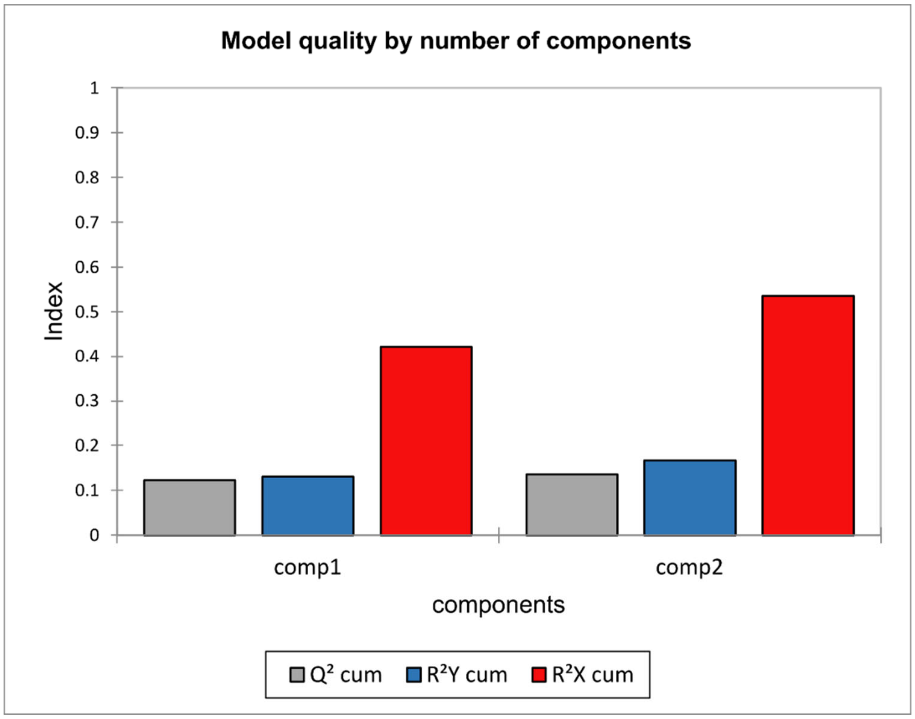

Figure S1. Model quality by number of components. Components were calculated using the Q^2(cum), R^2Y(cum), and R^2X(cum) parameters.

Table S3. Correlation matrix of the analyzed variables. CPUE = catch per unit effort, FCF = Fulton's condition factor, HSI = hepatosomatic index, PO = phenoloxidaze, proPO = prophenoloxidaze, THC = total hemocyte count.

VARIABLES	HSI	FCF	water temperature	CPUE
HSI	1	-0.207	-0.051	-0.113
FCF	-0.207	1	-0.232	0.154
water temperature	-0.051	-0.232	1	-0.835
CPUE	-0.113	0.154	-0.835	1
encapsulation response	0.054	-0.118	0.662	-0.680
THC	0.161	0.005	-0.038	-0.141
PO activity	0.018	-0.083	0.120	-0.071
total proPO	-0.207	-0.095	-0.237	0.215

Table S4. Principal component loadings (PC1 and PC2) on immune parameters of the invasive signal crayfish and the native narrow-clawed crayfish.

	**PC1**	**PC2**
Standard deviation	1.189	1.109
Proportion of Variance	0.353	0.307
Cumulative Proportion	0.353	0.661
encapsulation response	-0.240	-0.651
THC	-0.676	0.227
PO activity	0.159	-0.706
total proPO	-0.679	-0.162
